# Genomic Alterations and Tumor Mutation Burden in Merkel Cell Carcinoma

**DOI:** 10.1001/jamanetworkopen.2022.49674

**Published:** 2023-01-05

**Authors:** Danielle Brazel, Priyanka Kumar, Hung Doan, Tianyu Pan, Weining Shen, Ling Gao, Justin T. Moyers

**Affiliations:** 1 Department of Medicine, University of California, Irvine, Orange; 2 Unafilliliated Independent Contractor; 3 Department of Statistics, University of California, Irvine; 4 Department of Dermatology, Long Beach Veterans Health Administration, Long Beach, California; 5 Division of Hematology and Oncology, Department of Medicine, University of California, Irvine School of Medicine, Chao Family Comprehensive Cancer Center, Orange

## Abstract

**Importance:**

Merkel cell carcinoma (MCC) is a rare and highly aggressive cutaneous neuroendocrine carcinoma with increasing incidence. Cytotoxic chemotherapy and checkpoint inhibitors provide treatment options in the metastatic setting; however, there are no approved or standard of care targeted therapy treatment options.

**Objective:**

To identify actionable alterations annotated by the OncoKB database therapeutic evidence level in association with tumor mutation burden (TMB).

**Design, Setting, and Participants:**

This is a retrospective, cross-sectional study using data from the American Association for Cancer Research Genomics Evidence Neoplasia Information Exchange, a multicenter international cancer consortium database. Patients with MCC were enrolled in participating institutions between 2017 and 2022. Data from version 11.0 of the database were released in January 2022 and analyzed from April to June 2022.

**Main Outcomes and Measures:**

The main outcome was the percentage of patients with high TMB and OncoKB level 3B and 4 alterations.

**Results:**

A total of 324 tumor samples from 313 patients with MCC (107 women [34.2%]; 287 White patients [91.7%]; 7 Black patients [2.2%]) were cataloged in the database. The median (range) number of alterations was 4.0 (0.0-178.0), with a mean (SD) of 13.6 (21.2) alterations. Oncogenic alterations represented 20.2% of all alterations (862 of 4259 alterations). Tissue originated from primary tumor in 55.0% of patients (172 patients) vs metastasis in 39.6% (124 patients). TMB-high (≥10 mutations per megabase) was present in 26.2% of cases (82 patients). Next-generation sequencing identified 55 patients (17.6%) with a level 3B variation for a Food and Drug Administration–approved drug for use in a biomarker-approved indication or approved drug in another indication. An additional 8.6% of patients (27 patients) had a level 4 variation. Actionable alterations were more common among high TMB cases, with 37 of 82 patients (45.1%) harboring level 3 alterations compared with only 18 of 231 patients (7.8%) with low TMB. The most common level 3B gene variants included *PIK3CA* (12 patients [3.8%]), *BRCA1/2* (13 patients [4.2%]), *ATM* (7 patients [2.2%]), *HRAS* (5 patients [1.6%]), and *TSC1/2* (6 patients [1.9%]). The most common level 4 variants include *PTEN* (13 patients [4.1%]), *ARID1A* (9 patients [2.9%]), *NF1* (7 patients [2.2%]), and *CDKN2A* (7 patients [2.2%]). Copy number alterations and fusions were infrequent. In 61.0% of cases (191 cases), a PanCancer pathway was altered, and 39.9% (125 cases) had alterations in multiple pathways. Commonly altered pathways were *RTK-RAS* (119 patients [38.0%]), *TP53* (103 patients [32.9%]), cell cycle (104 patients [33.2%]), *PI3K* (99 patients [31.6%]), and *NOTCH* (93 patients [29.7%]). In addition, oncogenic DNA mismatch repair gene alterations were present in 8.0% of cases (25 patients).

**Conclusions and Relevance:**

In this cross-sectional retrospective study of alterations and TMB in MCC, a minority of patients had potentially actionable alterations. These findings support the investigation of targeted therapies as single agent or in combination with immunotherapy or cytotoxic chemotherapy in selected MCC populations.

## Key Points

**Question:**

Within a large multi-institutional genomic database, do tumors from patients with Merkel cell carcinoma stratified by tumor mutation burden (TMB) harbor actionable alterations?

**Findings:**

In this cross-sectional analysis of 324 tumor samples from 313 patients, 82 patients (26.2%) had a high TMB (≥10 mutations per megabase). Actionable alterations were more common among high TMB cases, with 37 of 82 patients (45.1%) harboring level 3 alterations compared with only 18 of 231 patients (7.8%) with low TMB.

**Meaning:**

These findings support the continued clinical investigation of targeted therapies as single agent or in combination with immunotherapy or cytotoxic chemotherapy in Merkel cell carcinoma.

## Teaser

This cross-sectional study analyzes genomic data to examine the presence of actionable alterations in Merkel cell carcinoma in association with tumor mutation burden.

## Introduction

Merkel cell carcinoma (MCC) is a highly aggressive cutaneous neuroendocrine carcinoma with incidence that has increased nearly 5-fold higher over recent decades.^1^ MCC is the second most common cause of death from skin cancer after melanoma, with a 5-year overall survival of 35% in nodal disease and 14% in the metastatic setting.^2^ It is a disease of elderly patients, with a median age at diagnosis of 76 years.^3^

The majority (80%) of MCCs harbor the tumorigenic DNA virus Merkel cell polyomavirus (MCPyV), which expresses oncogenic viral proteins.^4^ MCPyV-negative tumors generally have a higher tumor mutation burden (TMB) and worse prognosis than MCPyV-positive tumors.^5,6^ Combination cytotoxic chemotherapy (eg, carboplatin and etoposide) does not produce durable responses and is reserved for palliation of metastatic or refractory disease.^2^ Avelumab and pembrolizumab are both approved for advanced MCC with a 56% objective response rate and 24-month overall survival rate of 68.7% for first-line pembrolizumab.^7,8^ However, targeted therapies or immunotherapy combinations have yet to be approved in MCC.^9^

Given that many patients do not benefit from current treatments for MCC, targeted therapies have the potential to play an important role. We surveyed the presence of targetable alterations in MCC from the American Association for Cancer Research (AACR) Genomics Evidence Neoplasia Information Exchange (GENIE).

## Methods

The AACR Project GENIE database is a large, publicly accessible, international cancer registry that contains clinical data from 19 different participating cancer centers worldwide.^10^ Patient data were accessed from GENIE version 11.0, which was publicly released in January 2022 via cBioPortal, and were analyzed in May 2022. The present study analyzed publicly available deidentified data and was determined to be exempt from institutional review board review and the need for informed consent, in accordance with 45 CFR §46. This report follows the Strengthening the Reporting of Observational Studies in Epidemiology (STROBE) reporting guideline for retrospective cross-sectional studies.

Variables of interest extracted from the database included demographic data, genomic alterations with their OncoKB annotations for therapeutic evidence level, presence of The Cancer Genome Atlas PanCancer pathway alterations, and estimation of TMB.^11^ Demographic data collected for each patient included patient age at sequencing, sex, and race as recorded by the submitting institution. Race was analyzed in this study given the large variation in cancer incidence between races and the potential for differential variant factors by race. Recorded tumor characteristics included sample site (primary tumor vs metastases), total number of variants, number of oncogenic variants, number and type of structural variants, and number and type of copy number alterations (CNAs).

OncKB level of evidence (definitions are given in eTable 1 in Supplement 1) was recorded for variants, structural variants, and CNAs. OncoKB is a database of US Food and Drug Administration (FDA)–recognized genomic variants with evidence-based information about the level of actionability of these alterations.^12^ Variants were considered potentially actionable if they had an FDA-approved drug for use in a biomarker-approved indication or approved drug in another indication (levels 1-3). Level 4 evidence indicates potential targetability based on biological evidence.

### Statistical Analysis

Data were analyzed from April to June 2022 using SPSS statistical software version 28 (IBM). Categorical variables are presented as percentages and compared with χ^2^ tests. For continuous variable group comparisons, 2-sample *t* test and 2-sample proportion test are used. Two-sided *P* < .05 was considered statistically significant.

## Results

Of 136 096 samples present in AACR GENIE version 11.0, 1025 were nonmelanoma skin cancer samples that contained 324 MCC samples from 313 patients (107 women [34.2%]). Reported race was 91.7% White (287 patients), 2.2% Black (7 patients), and 0.6% Asian (2 patients). Full demographic data are presented in the Table.

**Table.** Demographic Data of Cohort and Key Findings of Genomic Alterations by Total Population and TMB Subgroup

Demographic data	Patients, No. (%)			*P* value
	Total population	TMB low (<10 mutations/Mb)	TMB high (≥10 mutations/Mb)	
Age at sequencing, y				
<40	6 (1.9)	5 (2.2)	1 (1.2)	.59
40-65	90 (28.8)	72 (31.2)	18 (21.9)	.11
66-79	146 (46.6)	107 (46.3)	39 (47.6)	.85
≥80	71 (22.7)	47 (20.3)	24 (29.3)	.10
Sex				
Male	206 (65.8)	144 (62.3)	62 (75.6)	.03
Female	107 (34.2)	87 (37.5)	20 (24.4)	.03
Race				
Asian	2 (0.6)	2 (0.9)	0	.40
Black	7 (2.2)	7 (3.0)	0	.11
White	287 (91.7)	208 (90.0)	79 (96.3)	.08
Unknown or not collected	17 (5.4)	14 (6.1)	3 (3.7)	.41
Sample type				
Primary	172 (54.9)	131 (56.7)	41 (50.0)	.29
Metastasis unspecified	115 (36.7)	83 (35.9)	32 (39.0)	.62
Distant organ metastasis	3 (1.0)	3 (1.3)	0	.30
Local recurrence	7 (2.2)	3 (1.3)	4 (4.9)	.06
Lymph node metastasis	6 (2.0)	5 (2.2)	1 (1.2)	.59
Not collected or unspecified	10 (3.2)	6 (2.6)	4 (4.9)	.31
Alterations classified as oncogenic, No./total No. (%)	862/4259 (20.2)	199/808 (24.6)	658/3451 (19.1)	<.001
Level 3B alterations present	55 (17.6)	18 (7.8)	37 (45.1)	<.001
Mean (range)	0.2 (0.0-4.0)	0.1 (0.0-3.0)	0.6 (0.0-4.0)	<.001
Level 3-4 alterations present	82 (26.2)	31 (13.4)	51 (62.2)	<.001
Mean (range)	0.4 (0.0-4.0)	0.2 (0.0-3.0)	1.0 (0.0-4.0)	<.001
Total alterations, median (range), No.	4.0 (0.0-178.0)	3.0 (0.0-20.0)	40.0 (1.0-178.0)	<.001
Oncogenic alterations, median (range), No.	1.0 (0.0-20.0)	0.0 (0.0-11.0)	7.5 (1.0-20.0)	<.001
The Cancer Genome Atlas pathways altered, mean (range), No.	2.2 (0.0-9.0)	0.8 (0.0-6.0)	6.0 (1.0-9.0)	<.001

Abbreviations: Mb, megabase; TMB, tumor mutation burden.

**Figure 1.**
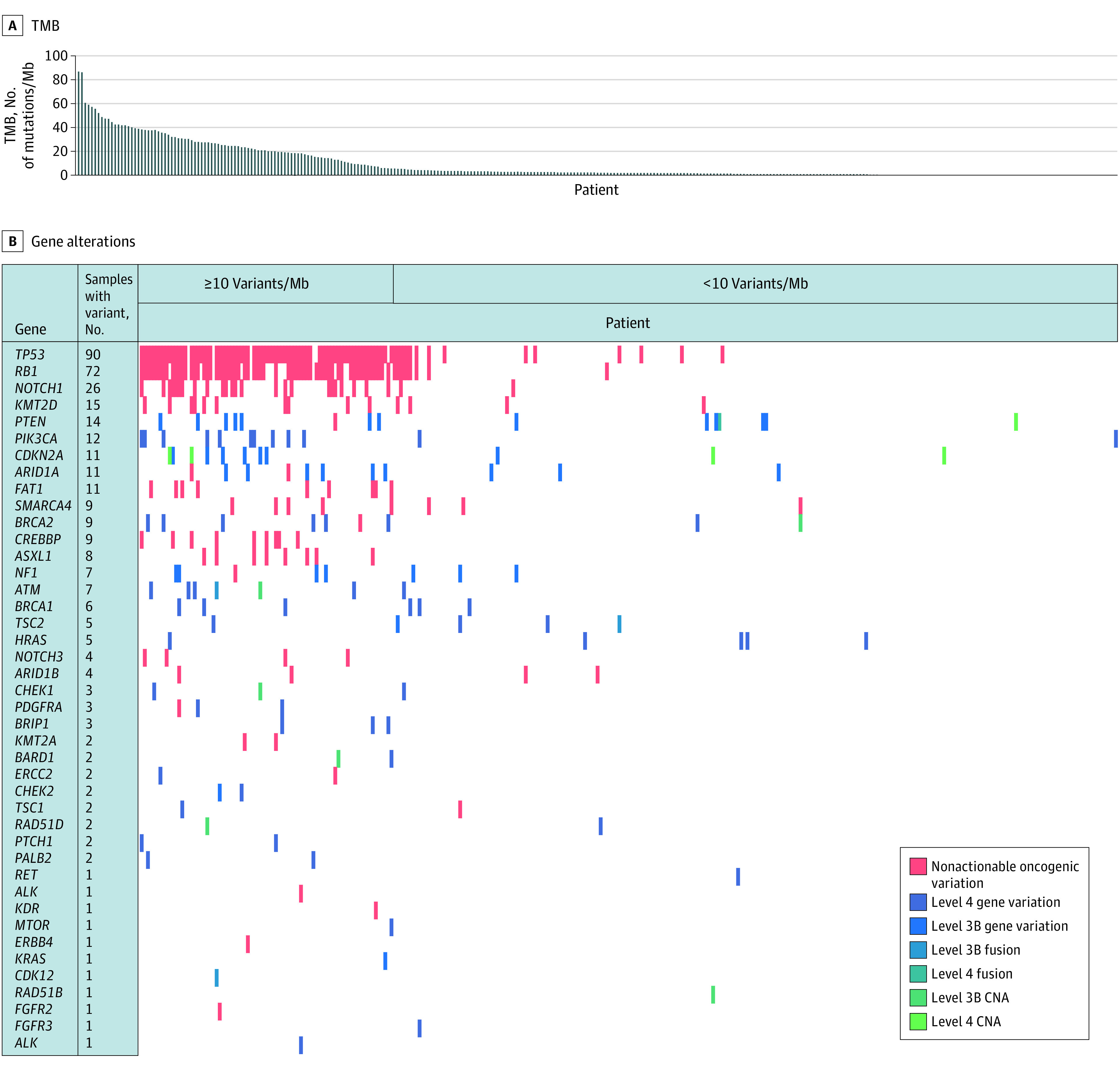
Genomic Alteration Heat Map A, Individual cases are presented by columns and arranged by tumor mutation burden (TMB) in descending order from left to right. B, Genes tested are presented as rows with each cell represented as no alteration (white) vs colored shades for nonactionable oncogenic alterations (red), gene variants (blue), fusions (blue-green), and copy number alteration (CNA) (green). Mb indicates megabase.

The median (range) number of alterations was 4.0 (0.0-178.0), and the mean (SD) was 13.6 (21.1) alterations. Oncogenic alterations represented 20.2% (862 of 4259 variants) of all variants. Tissue originated from primary tumor in 172 cases (55.0%) vs metastasis in 124 cases (39.6%). There are no FDA-approved targeted therapies for MCC; therefore, there are no level 1 or 2 alterations. Genomic sequencing identified 55 patients (17.6%) with an FDA-approved drug for use in a biomarker-approved indication or approved drug in another indication (level 3 variation). An additional 8.6% (27 patients) had a level 4 variation. The most common level 3B gene variants include *PIK3CA* (12 cases [3.8%]), *BRCA1/2* (13 cases [4.2%]), *ATM* (7 cases [2.2%]), *HRAS* (5 cases [1.6%]), and *TSC1/2* (6 cases [1.9%]). The most common level 4 variants include *PTEN* (13 cases [4.1%]), *ARID1A* (9 cases [2.9%]), *NF1* (7 cases [2.2%]), and *CDKN2A* (7 cases [2.2%]). Figure 1 shows a heat map in relation to TMB.

Only 3 fusions were identified: level 3B *ATM-CDK12* and intragenic* TSC2* and a level 4 intragenic *PTEN*. CNAs were identified in a small subset of patients. Level 3B CNAs included *ATM* (1 patient),* CHEK1* (1 patient), *BARD1 *(1 patient), *BRCA2* (1 patient), *RAD51B* (1 patient), and *RAD51D* (1 patient). Level 4 CNAs identified included *CDKN2A* (4 patients) and *PTEN* (1 patient).

Cases were separated into TMB cohorts of TMB high (TMB-H; ≥10 mutations per megabase) and TMB-low (TMB-L; <10 mutations per megabase). Within each cohort, 231 cases (73.8%) were TMB-L, whereas 82 cases (26.2%) were TMB-H. Among TMB-H cases, the most common level 3B alterations were *PIK3CA* (10 cases [12.2%]), *SMARCA4 *(6 cases [7.3%]),* NF1* (4 cases [4.9%]), *BRCA1* (3 cases [3.7%]), and *TSC1/2* (3 cases [3.7%]); the most common level 4 alterations were *PTEN* (7 cases [8.5%]), *CDKN2A* (6 cases [7.3%]), *ARID1A* (6 cases [5.3%]), and *ATM* (4 cases [4.9%]). Among TMB-L cases, the most common level 3B gene alterations were *BRCA1/2* (3 cases [1.3%]), *HRAS* (4 cases [1.7%]), *ARID1A* (2 cases [0.9%]), and *TSC1/2* (3 cases [1.3%]); the most common level 4 alterations were *PTEN* (6 cases [2.6%]) and *NF1* (2 cases [0.9%]). Actionable alterations were more common among TMB-H cases, with 37 of 82 patients (45.1%) harboring level 3 alterations compared with only 18 of 231 patients (7.8%) with TMB-L.

**Figure 2.**
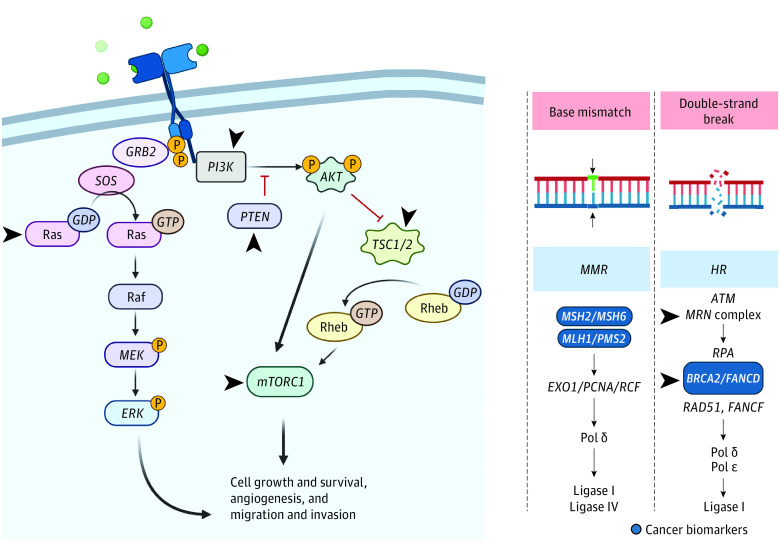
Frequently Altered Pathways in Merkel Cell Carcinoma (MCC) Data Set Figure was created in biorender.com. Arrowheads indicate frequently altered level 3 and 4 targets in MCC data set. Green shading denotes receptor ligands. Red Ts indicate inhibitor processes. *GDP* indicates guanosine diphosphate; *GTP*, guanosine triphosphate; P, phosphate.

In 61.0% of cases (191 cases), a PanCancer pathway was altered, and 125 cases (39.9%) had alterations in multiple pathways. Commonly altered pathways were *RTK-RAS* (119 cases [38.0%]), *TP53* (103 cases [32.9%]), cell cycle (104 cases [33.2%]), *PI3K* (99 cases [31.6%]), and *NOTCH* (93 cases [29.7%]) (Figure 2 and eFigure in Supplement 1). In addition, oncogenic DNA mismatch repair gene alterations were present in 25 cases (8.0%).

## Discussion

In this cross-sectional analysis of 324 samples from 313 patients, to our knowledge, we present the largest genomic analysis of MCC patient samples to date. We found 20.2% of alterations identified to be oncogenic. Variants that were potentially targetable with an FDA-approved drug were present in 17.6% of patients (55 patients), and 61.0% of cases had a PanCancer pathway altered.

Many of the most frequent actionable alterations within TMB-H tumors were within tumor suppressor pathways (*PIK3CA/PTEN*, *CDKN2A*, *BRCA1/2*, *NF1*, *ATM*, and *TSC1/2*), suggesting that many variants may be passenger rather than driver alterations in the setting of highly altered tumors. However, there remains a minority of patients with TMB-L and TMB-H tumors who have actionable and potentially actionable alterations.

Previously, single institution and small case series have described smaller sets of genomic analysis from patients with MCC.^13,14,15,16,17,18,19^ A review by Erstad et al^13^ noted that the most common variant genes in patients with MCC included *RB* (a restrictor of the cell cycle), *TP53*, and *PIK3CA*. In a small set of tumors, Harms et al^14^ showed that MCPyV-negative tumors were TMB-H and had an ultraviolet signature with additional oncogenic alterations in *HRAS*, *PRUNE2*, and *NOTCH* family genes, whereas MCPyV-positive tumors were TMB-L and had no ultraviolet signature. Similarly, Wong and colleagues^15^ analyzed 34 patients with a 619-gene panel and found that all virus-negative tumors harbored *RB1* or *TP53* variants with an increased frequency of *NOTCH1* and *FAT1* variants. *MAPK* and *PI3K* pathway alterations were also common. In a single-institution study of 17 patients by Cohen et al,^20^ there was a high frequency of variants in the *TP53* gene (12 of 17 cases [71%]); cell cycle pathway (*CDKN2A/B*, *CDKN2C*, or *RB1*; 12 of 17 cases [71%]); *PI3K*, *AKT*, and *mTOR* pathway (9 of 17 cases [53%]); and DNA repair genes (5 of 17 cases [29%]). Although the small sample size limited generalizability, they found frequencies of variants similar to those we observed.

The only study of comparable size to ours is from a single next-generation sequencing platform analysis of 317 tumors.^21^ Using known genomic sequences of MCPyV, the authors were able to separate MCPyV-positive vs MCPyV-negative tumors and TMB-H (≥20 mutations per megabase; 117 cases) vs TMB-L (≤20 mutations per megabase; 175 cases) status.^21^ The most common variants in that cohort were *TP53*, *RB1*, *NOTCH1*, *KMT2D*, and *FAT1*, with an incidence of more than 25% among TMB-H MCCs.^21^ The most frequent mutations in TMB-L MCCs were the same, but no variation had an incidence greater than 10%.^21^ Notably, that study did not report the actionability of variants.^21^

Although targeted therapy and immunotherapy combinations have been successful in other cancer types, MCC has been infrequently included within targeted therapy basket trials.^22^ These results reveal that targeted therapies may be effective in select patients with variants in commonly altered pathways, including the *TP53*, cell cycle, *PI3KA*, and *RTK-RAS* pathways. Ongoing and reported clinical trials using targeted therapies are shown in eTable 2 in Supplement 1.

### Limitations

This analysis is limited by database constraints, and bias may exist in terms of which samples are submitted for including by participating institutions. Variables not captured by the database included cancer stage, systemic and surgical treatments and outcomes, and the presence of MCPyV. Nonuniform next-generation sequencing testing panels lead to variation in tested genes and reporting of zygosity, copy numbers, and allele fraction.

## Conclusions

This cross-sectional study found that most patients with MCC had an oncogenic alteration in a cancer pathway and identified a subset of patients with targetable variants in MCC. However, the majority of targetable variants occurred in TMB-H tumors. These findings may support the investigation of small molecule inhibitors as single agent or in combination with immunotherapy or cytotoxic chemotherapy in MCC.

## Notes

Supplement 1. **eTable 1.** OncoKB Therapeutic Levels of Evidence **eFigure.** Percentage of Patients With Each of the 10 TCGA PanCancer Pathways Altered **eTable 2.** Summary of Ongoing Published and Registered Trials From NCT.gov Utilizing Targeted Therapy Clinical Trials in Merkel Cell Carcinoma
eReferences

Supplement 2.
Data Sharing Statement

## References

[1] Harms KL, Lazo de la Vega L, Hovelson DH, . Molecular profiling of multiple primary Merkel cell carcinoma to distinguish genetically distinct tumors from clonally related metastases. JAMA Dermatol. 2017;153(6):505-512. doi:10.1001/jamadermatol.2017.0507 28403382 PMC5540059

[2] Schadendorf D, Lebbé C, Zur Hausen A, . Merkel cell carcinoma: epidemiology, prognosis, therapy and unmet medical needs. Eur J Cancer. 2017;71:53-69. doi:10.1016/j.ejca.2016.10.022 27984768

[3] Harms KL, Healy MA, Nghiem P, . Analysis of prognostic factors from 9387 Merkel cell carcinoma cases forms the basis for the new 8th edition AJCC staging system. Ann Surg Oncol. 2016;23(11):3564-3571. doi:10.1245/s10434-016-5266-4 27198511 PMC8881989

[4] Feng H, Shuda M, Chang Y, Moore PS. Clonal integration of a polyomavirus in human Merkel cell carcinoma. Science. 2008;319(5866):1096-1100. doi:10.1126/science.1152586 18202256 PMC2740911

[5] Paulson KG, Lemos BD, Feng B, . Array-CGH reveals recurrent genomic changes in Merkel cell carcinoma including amplification of L-Myc. J Invest Dermatol. 2009;129(6):1547-1555. doi:10.1038/jid.2008.365 19020549 PMC2830552

[6] Tetzlaff MT, Nagarajan P. Update on Merkel cell carcinoma. Head Neck Pathol. 2018;12(1):31-43. doi:10.1007/s12105-018-0898-2 29556962 PMC5873498

[7] D’Angelo SP, Bhatia S, Brohl AS, . Avelumab in patients with previously treated metastatic Merkel cell carcinoma: long-term data and biomarker analyses from the single-arm phase 2 JAVELIN Merkel 200 trial. J Immunother Cancer. 2020;8(1):e000674. doi:10.1136/jitc-2020-000674 32414862 PMC7239697

[8] Nghiem P, Bhatia S, Lipson EJ, . Durable tumor regression and overall survival in patients with advanced Merkel cell carcinoma receiving pembrolizumab as first-line therapy. J Clin Oncol. 2019;37(9):693-702. doi:10.1200/JCO.18.01896 30726175 PMC6424137

[9] Yap TA, Parkes EE, Peng W, Moyers JT, Curran MA, Tawbi HA. Development of immunotherapy combination strategies in cancer. Cancer Discov. 2021;11(6):1368-1397. doi:10.1158/2159-8290.CD-20-1209 33811048 PMC8178168

[10] AACR Project GENIE Consortium. AACR Project GENIE: powering precision medicine through an international consortium. Cancer Discov. 2017;7(8):818-831. doi:10.1158/2159-8290.CD-17-0151 28572459 PMC5611790

[11] Anaya J, Sidhom J-W, Cummings CA, Baras AS; AACR Project GENIE Consortium. Aggregation tool for genomic concepts (ATGC): a deep learning framework for sparse genomic measures. bioRxiv. Preprint posted online November 8, 2021. doi:10.1101/2020.08.05.237206

[12] Chakravarty D, Gao J, Phillips SM, . OncoKB: a precision oncology knowledge base. JCO Precis Oncol. 2017;1:PO.17.00011. doi:10.1200/PO.17.00011 28890946 PMC5586540

[13] Erstad DJ, Cusack JC Jr. Mutational analysis of Merkel cell carcinoma. Cancers (Basel). 2014;6(4):2116-2136. doi:10.3390/cancers6042116 25329450 PMC4276959

[14] Harms PW, Vats P, Verhaegen ME, . The distinctive mutational spectra of polyomavirus-negative Merkel cell carcinoma. Cancer Res. 2015;75(18):3720-3727. doi:10.1158/0008-5472.CAN-15-0702 26238782 PMC4573907

[15] Wong SQ, Waldeck K, Vergara IA, . UV-associated mutations underlie the etiology of MCV-negative Merkel cell carcinomas. Cancer Res. 2015;75(24):5228-5234. doi:10.1158/0008-5472.CAN-15-1877 26627015

[16] Goh G, Walradt T, Markarov V, . Mutational landscape of MCPyV-positive and MCPyV-negative Merkel cell carcinomas with implications for immunotherapy. Oncotarget. 2016;7(3):3403-3415. doi:10.18632/oncotarget.6494 26655088 PMC4823115

[17] González-Vela MDC, Curiel-Olmo S, Derdak S, . Shared oncogenic pathways implicated in both virus-positive and UV-induced Merkel cell carcinomas. J Invest Dermatol. 2017;137(1):197-206. doi:10.1016/j.jid.2016.08.015 27592799

[18] Carter MD, Gaston D, Huang WY, . Genetic profiles of different subsets of Merkel cell carcinoma show links between combined and pure MCPyV-negative tumors. Hum Pathol. 2018;71:117-125. doi:10.1016/j.humpath.2017.10.014 29079179

[19] Nardi V, Song Y, Santamaria-Barria JA, . Activation of PI3K signaling in Merkel cell carcinoma. Clin Cancer Res. 2012;18(5):1227-1236. doi:10.1158/1078-0432.CCR-11-2308 22261808 PMC3912509

[20] Cohen PR, Tomson BN, Elkin SK, Marchlik E, Carter JL, Kurzrock R. Genomic portfolio of Merkel cell carcinoma as determined by comprehensive genomic profiling: implications for targeted therapeutics. Oncotarget. 2016;7(17):23454-23467. doi:10.18632/oncotarget.8032 26981779 PMC5029639

[21] Knepper TC, Montesion M, Russell JS, . The genomic landscape of Merkel cell carcinoma and clinicogenomic biomarkers of response to immune checkpoint inhibitor therapy. Clin Cancer Res. 2019;25(19):5961-5971. doi:10.1158/1078-0432.CCR-18-4159 31399473 PMC6774882

[22] Horak P, Heining C, Kreutzfeldt S, . Comprehensive genomic and transcriptomic analysis for guiding therapeutic decisions in patients with rare cancers. Cancer Discov. 2021;11(11):2780-2795. doi:10.1158/2159-8290.CD-21-0126 34112699

