# Supplementary material for: Genomic Alterations and Tumor Mutation Burden in Merkel Cell Carcinoma
Source: JAMA Netw Open. 2023 Jan 5;6(1):e2249674. doi: 10.1001/jamanetworkopen.2022.49674 (PMC9856969; doi:10.1001/jamanetworkopen.2022.49674)
Supplement: Supplement 1. — eTable 1. OncoKB Therapeutic Levels of Evidence eFigure. Percentage of Patients With Each of the 10 TCGA PanCancer Pathways Altered eTable 2. Summary of Ongoing Published and Registered Trials From NCT.gov Utilizing Targeted Therapy Clinical Trials in Merkel Cell Carcinoma eReferences [file jamanetwopen-e2249674-s001.pdf]

Supplemental Online Content

Brazel D, Kumar P, Doan H, et al. Genomic alterations and tumor mutation burden in Merkel cell carcinoma. *JAMA Netw Open*. 2023;6(1):e2249674. doi:10.1001/jamanetworkopen.2022.49674

eTable 1. OncoKB Therapeutic Levels of Evidence
eFigure. Percentage of Patients With Each of the 10 TCGA PanCancer Pathways Altered
eTable 2. Summary of Ongoing Published and Registered Trials From NCT.gov Utilizing Targeted Therapy Clinical Trials in Merkel Cell Carcinoma
eReferences

This supplemental material has been provided by the authors to give readers additional information about their work.

© 2023 Brazel D et al. *JAMA Network Open.*

eTable 1: OncoKB Therapeutic Levels of Evidence

Level of Evidence	Definition
L1	There is an FDA-biomarker that predicts a response to an FDA-approved drug for this indication
L2	Indicates a standard care biomarker recommended by the NCCN or other professional guidelines that predict a response to an FDA-approved drug for this indication
L3A	Indicates compelling clinical evidence supports the biomarker as being predictive of response to a drug in this indication
L3A	Indicates a standard care or investigational biomarker predictive of response to an FDA-approved or investigational drug in another indication
L4	Indicates compelling biological evidence supports the biomarker as being predictive of response to a drug

Adapted from oncokb.com

© 2023 Brazel D et al. *JAMA Network Open.*

eFigure: Percentage of Patients With Each of the 10 TCGA PanCancer Pathways Altered

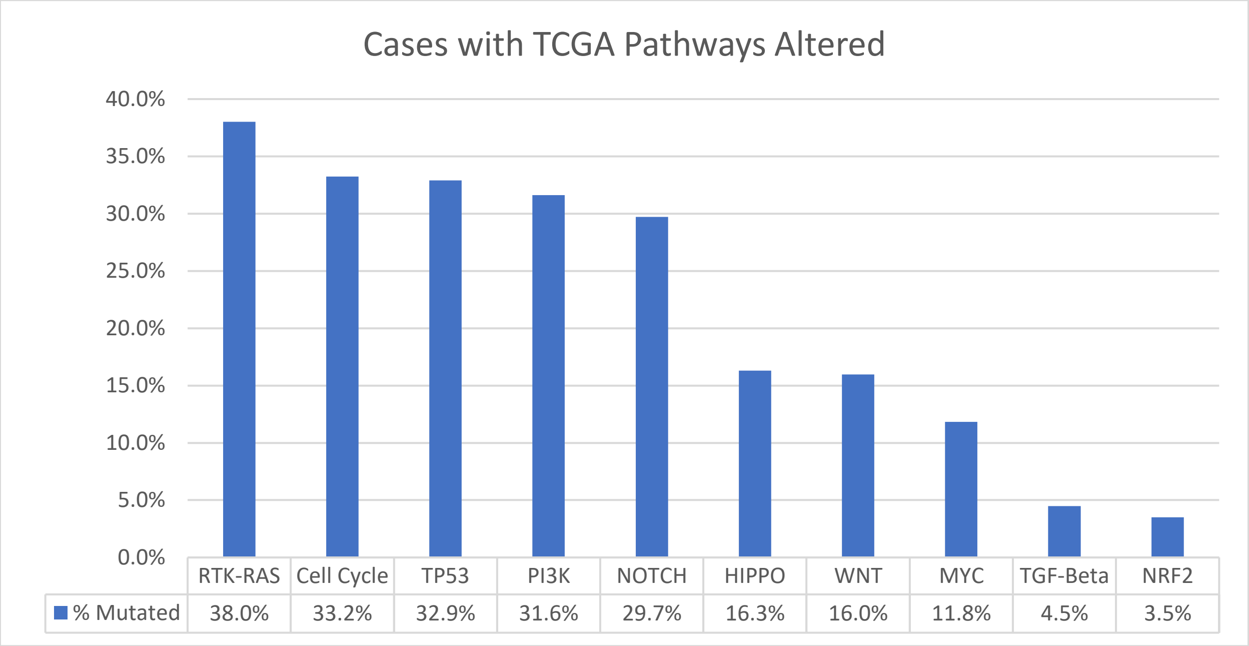

© 2023 Brazel D et al. *JAMA Network Open.*

eTable 2: Summary of Ongoing Published and Registered Trials From NCT.gov Utilizing Targeted Therapy Clinical Trials in Merkel Cell Carcinoma

NCT	Phase	Cancer Types	Drug(s)	Targeted Therapy Mechanism	ORR	Status	Site	Ref
NCT03787602	1b/2	P53 Wild Type PD-1 refractory MCC	Navtemadlin (KRT-232)	MDM2 inhibitor	33% (3/9)	Recruiting	Multiple Institutions, US	[1,2]
NCT02036476	2	Recurrent or Metastatic MCC	Cabozatanib	Multiple receptor TKI	0% (0/8)	Active, not recruiting	Dana Farber Cancer Institute	[3]
NCT10125877	2	Metastatic MCC	Pazopanib	Multiple receptor TKI	19% (3/16)	Completed	Multiple institutions, international	[4]
NCT02514824	1/2	Recurrent or Metastatic MCC	MLN0128	mTOR inhibitor	NYR	Closed	Dana Farber Cancer Institute	
NCT04393753 (MERKLIN2)	2	PD-1 refractory MCC	Avelumab + Domatinostat	Histone deacetylase inhibitor	NYR	Active, not recruiting	Multiple institutions, international	
NCT04869137	2	Neoadjuvant treatment in Resectable MCC	Lenvatinib + Pembrolizumab	Multiple receptor TKI	NYR	Recruiting	Moffitt Cancer Center	
NCT00068783 (S0331)	2	Unresectable or Metastatic MCC	Imatinib	BCR/ABL TKI	4% (1/23)	Completed	Southwest Oncology Group	[5]
NCT00079131	2	Metastatic or Recurrent MCC	Oblimersen	Bcl-2 antisense phosphorothioate oligonucleotide	NYR	Completed	Memorial Sloan-Kettering	

ORR= Objective Response Rate (complete response + partial responses)
NYR= Not yet reported
TKI= Tyrosine Kinase Inhibitor

© 2023 Brazel D et al. *JAMA Network Open.*

eReferences

1. Wong MKK, Kelly CM, Burgess MA, et al. KRT-232, a first-in-class, murine double minute 2 inhibitor (MDM2i), for TP53 wild-type (p53WT) Merkel cell carcinoma (MCC) after anti–PD-1/L1 immunotherapy. *Journal of Clinical Oncology*. 2020;38(15_suppl):10072-10072. doi:10.1200/JCO.2020.38.15_suppl.10072
2. Wong MKK, Burgess MA, Chandra S, et al. Navtemadlin (KRT-232) activity after failure of anti-PD-1/L1 therapy in patients (pts) with TP53WT Merkel cell carcinoma (MCC). *Journal of Clinical Oncology*. 2022;40(16_suppl):9506-9506. doi:10.1200/JCO.2022.40.16_suppl.9506
3. Rabinowits G, Lezcano C, Catalano PJ, et al. Cabozantinib in Patients with Advanced Merkel Cell Carcinoma. *Oncologist*. Jul 2018;23(7):814-821. doi:10.1634/theoncologist.2017-0552
4. Nathan PD, Gaunt P, Wheatley K, et al. UKMCC-01: A phase II study of pazopanib (PAZ) in metastatic Merkel cell carcinoma. *Journal of Clinical Oncology*. 2016;34(15_suppl):9542-9542. doi:10.1200/JCO.2016.34.15_suppl.9542
5. Samlowski WE, Moon J, Tuthill RJ, et al. A phase II trial of imatinib mesylate in merkel cell carcinoma (neuroendocrine carcinoma of the skin): A Southwest Oncology Group study (S0331). *Am J Clin Oncol*. Oct 2010;33(5):495-9. doi:10.1097/COC.0b013e3181b9cf04

© 2023 Brazel D et al. *JAMA Network Open.*
