# Supplementary material for: Genomic Alterations and Tumor Mutation Burden in Merkel Cell Carcinoma
Source: JAMA Netw Open. 2023 Jan 5;6(1):e2249674. doi: 10.1001/jamanetworkopen.2022.49674 (PMC9856969; doi:10.1001/jamanetworkopen.2022.49674)
Supplement: Supplement 2. — Data Sharing Statement [file jamanetwopen-e2249674-s002.pdf]

# Data Sharing Statement

Brazel. Genomic Alterations and Tumor Mutation Burden in Merkel Cell Carcinoma. *JAMA Netw Open*. Published January 05, 2023. doi:10.1001/jamanetworkopen.2022.49674

**Data**
**Data available:** Yes
**Data types:** Deidentified participant data
**How to access data:** https://genie.cbioportal.org/login.jsp
**When available:** With publication

**Supporting Documents**
**Document types:** None

**Additional Information**
**Who can access the data:** anyone requesting the data from cbiioportal
**Types of analyses:** for any purpose
**Mechanisms of data availability:** access by investigator
